# Outpatient ambulatory EEG as an option for epilepsy surgery evaluation instead of inpatient EEG telemetry^☆^

**DOI:** 10.1016/j.ebcr.2013.01.001

**Published:** 2013-03-06

**Authors:** Syed A. Rizvi, José F. Téllez Zenteno, Sara L. Crawford, Adam Wu

**Affiliations:** a Division of Neurology, Department of Medicine, Royal University Hospital, University of Saskatchewan, Saskatoon, Saskatchewan, Canada; b Clinical Neurophysiology Laboratory, Royal University Hospital, Saskatoon, Canada; c Division of Neurosurgery, Department of Surgery, Royal University Hospital, University of Saskatchewan, Saskatoon, Saskatchewan, Canada

**Keywords:** Ambulatory EEG, Portable EEG, Epilepsy, Complex partial seizure, Diagnostic yield, Cost-effectiveness

## Abstract

Outpatient ambulatory EEG is more cost-effective than inpatient EEG telemetry and may provide adequate seizure localization in a presurgical evaluation. A 51-year-old right-handed male had been unable to work or drive since the age of 35 due to intractable partial onset epilepsy. A 72-hour outpatient ambulatory EEG recorded 18 seizures from the right temporal region. No epileptiform activity was seen in the left hemisphere. Magnetic resonance imaging showed right mesial temporal sclerosis as well as an area of encephalomalacia at the medial inferior right temporal lobe. Neuropsychological assessment found that the patient was a good neurosurgery candidate. At this point, the patient was considered to be a candidate for a right temporal lobectomy. A standard right temporal lobectomy was performed. The patient has been seizure-free for 10 months after the surgery. Follow-up EEGs show no epileptiform activity. The patient is preparing to go back to work, and his driver's license was reinstated 9 months postsurgery. Neuropsychological reassessment is pending, but no apparent change in cognition has been noticed by the patient or his family. Cases with a high congruence between diagnostic imaging and the EEG abnormalities identified in the portable EEG may provide enough information regarding seizure frequency and localization to eliminate the need for inpatient EEG telemetry in the evaluation of patients for epilepsy surgery. We believe that the use of aEEG in preoperative planning should be restricted to cases of TLE and to patients with a high frequency of seizures.

## 1 Introduction

The electroencephalogram (EEG) is central to the diagnosis of epilepsy [1], [2], [3]. Seizures may manifest in the EEG along with interictal events, the combination of which provides useful information in the diagnosis and localization of epileptic brain loci. However, seizures can be rare events so capturing them on a short EEG recording is difficult. The trend has shifted in favor of prolonged EEG recordings [1], [2]. While long-term inpatient video-EEG (vEEG) telemetry has long been considered the gold standard for preoperative evaluation of patients with epilepsy, it is resource-intensive, time-consuming, costly, and not universally available [3].

Ambulatory EEG (aEEG) enables EEG recording on a portable unit while the patient conducts normal activities of life at home, school, or work. There is concern that the quality of aEEG recordings may be more susceptible to artifact degradation and electrode connection issues as the patient is unmonitored and not confined to a single location. Nevertheless, aEEG is clinically useful in 75% of tested patients, with abnormalities found in 12–25% of patients in whom the initial EEG was normal or nondiagnostic. Moreover, aEEG recording costs approximately 50% of traditional inpatient monitoring [4].

In a departure from routine, we report a patient who underwent a right temporal lobectomy based on suggestive clinical semiology correlated with an epileptiform focus identified on magnetic resonance imaging (MRI) and a solitary outpatient ambulatory EEG study.

## 2 Material and methods

A 51-year-old right-handed man initially presented with a generalized tonic-clonic seizure at the age of 25 at which time treatment with carbamazepine was instituted. He remained seizure-free until the age of 35 when he began to experience daily complex partial seizures characterized by staring spells, lip smacking, and loss of awareness lasting 30–90 s. He did not report any kind of subjective sensation preceding seizure onset. The seizures were sufficiently disabling so as to preclude employment. Seizure control could not be achieved with antiepileptic medication despite trials with 8 standard agents alone or in combination.

## 3 Results

**Fig. 1:**
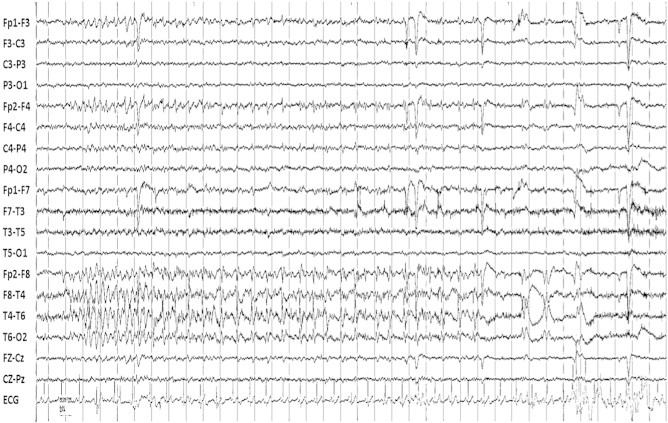
One of the 18 seizures recorded with the aEEG. The seizure is characterized by rhythmic sharp theta mixed with spikes over the electrodes F8–T4 with spread to Fp2 and T6. The seizure finishes with sharp waves at F8 and T4 and delta. All the seizures were similar.

The aEEGs were recorded using 24 AC channels with 4 differential and 4 auxiliary DC channels capable of continuous recording (XLTEK Trex Ambulatory System). Gold-plated cup EEG electrodes with a 10-mm diameter and a 2-mm center hole were attached to the scalp with collodion, according to the International 10–20 System. The 72-hour outpatient aEEG recording identified 18 electrographic seizure events originating from the right temporal focus (Fig. 1). Seizure onset was characterized by rhythmic theta activity mixed with spikes at the electrodes T4–F8 with spread to T6, followed by 4- to 5-Hz spike-and-wave discharge activity in the same region. The total duration ranged from 20 to 70 s. Right temporal theta and delta slowing was observed in the postictal phase. Interictal spikes were also present in the right temporal region, with the maximum at T4. No epileptiform activity was evident in the left hemisphere.

**Fig. 2:**
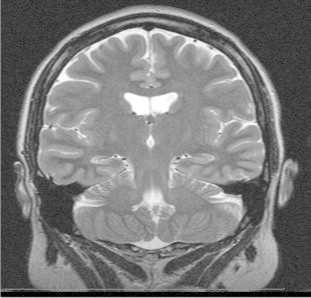
A coronal section shows clear atrophy of the right hippocampus with signal changes consistent with mesial temporal sclerosis.

At the time of evaluation, the patient was taking lamotrigine (150 mg twice daily), valproic acid (500 mg three times per day), and carbamazepine (400 mg in the morning and 600 mg in the evening). Magnetic resonance imaging of the brain disclosed right mesial temporal sclerosis (Fig. 2) as well as an area of encephalomalacia at the medial inferior right temporal lobe. Neuropsychological assessment indicated that the patient was cognitively intact. Typically, vEEG monitoring documentation of seizure localization has been considered as one of the most important aspects of a presurgical investigation in refractory temporal lobe epilepsy (TLE). Patients are not routinely considered for surgical resection until inpatient vEEG telemetry is performed. In this case, however, the aEEG was strongly suggestive of a right medial temporal seizure focus. This observation was further supported by homologous clinical and neuroimaging data. The patient was thus considered to be an ideal candidate for a right temporal lobe resection, even in the absence of confirmatory inpatient vEEG telemetry.

## 4 Results

A standard right temporal lobectomy was performed. The patient has remained seizure-free 10 months after surgery. Follow-up EEGs have demonstrated right temporal 2- to 3-Hz polymorphic delta, but no epileptiform activity has been identified. At last follow-up, valproic acid had been discontinued, and the patient was maintained on the same preoperative doses of lamotrigine and carbamazepine. The patient's driver's license was reinstated 9 months postsurgery, and he is now gainfully employed. Neuropsychological assessment indicated stable cognitive function with no evidence of decline.

## 5 Discussion

Although the efficacy of aEEG versus inpatient alternatives has been reported in some studies [5], [6], [7], [8], [9], [10], [11], we could not locate any published literature on the utilization of a single 72-hour aEEG recording as the sole electrophysiological criterion for ictal locus identification and subsequent resection. This option is substantially more cost-effective than its inpatient counterparts, safe, and readily available, and it may obviate the need for intensive inpatient vEEG monitoring in select patients [3], [12], [13], [14]. A further advantage of aEEG may be that spontaneous seizure frequency appears to decrease in patients with drug-refractory epilepsy on admission to a monitoring unit [14].

In select patients, the combination of clinical and outpatient aEEG findings is sufficient to plan surgical therapy [13], [14], [15]. Chang et al. reviewed the data of 7 temporal lobectomy patients whose preoperative monitoring was performed entirely outside the hospital. The mean baseline seizure frequency was at least 9.1 seizures/week with an average of 7.4 seizures recorded over 9.4 days of monitoring. Only 1 patient had any antiepileptic drug taper; none suffered any complications following temporal lobectomy on the side of demonstrated ictal onset. Postoperative follow-up averaged 5.5 years, and all patients were either seizure-free or had only rare disabling or nocturnal seizures (4 patients had outcomes in Engel's class I and 3 patients in Engel's class II). A comparison group who underwent standard inpatient monitoring was similar in average seizure frequency, monitoring duration, number of seizures recorded, and postoperative outcome, although all but one had antiepileptic drugs tapered off during monitoring [14].

In 1983, Ebersole and Leroy conducted the only study comparing aEEG to vEEG. In a sample of 40 children, accordant diagnosis for normal EEG was 100%, with 60% for abnormal nonepileptic EEG and 54% for abnormal epileptic EEG. Correct lateralization and anterior versus posterior localization of epileptiform features occurred in 78% and 72% of the sample, respectively. However, aEEG recordings were obtained using only 3 channels which likely limited the interpretation [6].

In the absence of high-quality comparative data, indications for aEEG in the preoperative assessment for patients with epilepsy remain empirical [13], [14]. We believe that the use of aEEG in preoperative planning should be restricted to cases of TLE and to patients with a high frequency of seizures [13]. To be useful for epilepsy surgery decisions, an aEEG recording must show an adequate number of seizures and interictal abnormalities. These findings should be highly congruent with the rest of the investigations that include neuropsychology and imaging modalities [9]. Only a few case reports have addressed the use of aEEG for extratemporal cases of epilepsy. Further research is required to more fully explore the usefulness of this modality [13], [14].

## 6 Conclusion

In patients with TLE in whom there is a clear congruence of diagnostic imaging and EEG findings, outpatient ambulatory EEG may provide sufficient information regarding seizure frequency and localization so as to obviate the need for inpatient EEG telemetry in the evaluation of patients for epilepsy surgery.

## Disclosures

Dr. Tellez receives grants from the University of Saskatchewan and the Royal University Hospital Foundation, Saskatoon, Saskatchewan, through the Mudjadik Thyssen Mining Professorship in Neurosciences.

Dr. Syed Rizvi has no association with any corporation or foundation.

Ms. Sara Crawford has no association with any corporation or foundation.
